# Supplementary material for: Identification of photoperiod-induced specific miRNAs in the adrenal glands of Sunite sheep (Ovis aries)
Source: Front Vet Sci. 2022 Jul 22;9:888207. doi: 10.3389/fvets.2022.888207 (PMC9354845; doi:10.3389/fvets.2022.888207)
Supplement: Supplementary file 1 [file Data_Sheet_1.ZIP › Supplementary file/miRDeep2 file.zip!/miRDeep2 file/Know_Structure/SPLP42A1/oar-let-7d.pdf]

miRBase precursor	: oar-let-7d
Total read count	: 26385
oar-let-7d read count	: 24336
remaining reads	: 2049

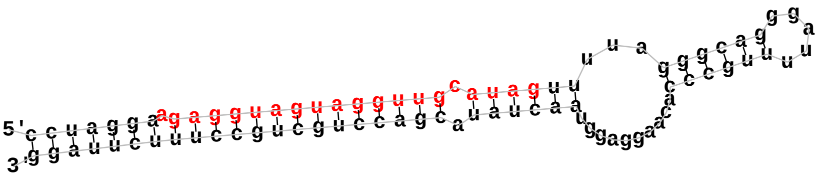

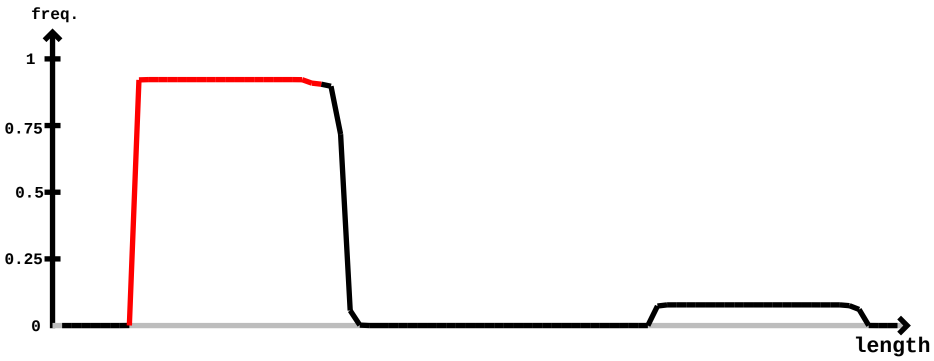


```
        oar-let-7d
5'- ccuaggaagagguaguagguugcauaguuuuagggcagggauuuugcccacaaggagguaacuauacgaccugcugccuuucuuagg  -3'    exp
    (((((((.((((((((((((((.((((((...((((((.....))))))..........)))))).)))))))))))))))))))))    reads      mm         sample
    ......aagagguaguagguugcau..............................................................    1          0          seq
    ......aagagguaguagguugcauagu...........................................................    3          0          seq
    ......aagagguaguagguugcauaguu..........................................................    5          0          seq
    .......agagguaguagguugca...............................................................    11         0          seq
    .......agaggCaguagguugcau..............................................................    1          1          seq
    .......agagguaguagguugcau..............................................................    335        0          seq
    .......agagguaguagguugcaua.............................................................    108        0          seq
    .......agagguaguagguugcauag............................................................    200        0          seq
    .......agagguaguagguugcauagu...........................................................    4742       0          seq
    .......agagguaguagguugcauaguu..........................................................    17415      0          seq
    .......agagguaguagguugcauaguuu.........................................................    1439       0          seq
    .......agagguaguagguugcauaguuuu........................................................    45         0          seq
    .......agagguaguagguugcauaguuuua.......................................................    1          0          seq
    ........gagguaguagguugcau..............................................................    1          0          seq
    ........gagguaguagguugcauaguu..........................................................    26         0          seq
    ........gagguaguagguugcauaguuu.........................................................    2          0          seq
    .........agguaguagguugcauagu...........................................................    1          0          seq
    .............................uuagggcagggauuuugcccacaaggaggu............................    2          0          seq
    .......................................................agguaacuauacgaccugcugccuuuc.....    1          0          seq
    ..........................................................uaacuauacgaccugcugccuuu......    1          0          seq
    ..........................................................uaacuauacgaccugcugccuuuc.....    1          0          seq
    .............................................................cuauacgaccugcugcc.........    1          0          seq
    .............................................................cuauacgaccugcugccuu.......    2          0          seq
    .............................................................cuauacgaccugcugccuuu......    71         0          seq
    .............................................................cuauacgaccugcugccuuuc.....    348        0          seq
    .............................................................cuauacgaccugcugccuuucu....    1500       0          seq
    .............................................................cuauacgaccugcugccuuucuu...    7          0          seq
    ..............................................................uauacgaccugcugccuuu......    6          0          seq
    ..............................................................uauacgaccugcugccuuuc.....    19         0          seq
    ..............................................................uauacgaccugcugccuuucu....    89         0          seq
    ...............................................................auacgaccugcugccuuucu....    1          0          seq
```
